# HIV Patients Developing Primary CNS Lymphoma Lack EBV-Specific CD4^+^ T Cell Function Irrespective of Absolute CD4^+^ T Cell Counts

**DOI:** 10.1371/journal.pmed.0040096

**Published:** 2007-03-27

**Authors:** Olivier Gasser, Florian K Bihl, Marcel Wolbers, Elisabetta Loggi, Ingrid Steffen, Hans H Hirsch, Huldrych F Günthard, Bruce D Walker, Christian Brander, Manuel Battegay, Christoph Hess, for the Swiss HIV Cohort Study

**Affiliations:** 1 Immunobiology Laboratory, Department of Research, University Hospital Basel, Basel, Switzerland; 2 Partners AIDS Research Center, Massachusetts General Hospital, Boston, Massachusetts, United States of America; 3 Institute for Clinical Epidemiology, University Hospital Basel, Basel, Switzerland; 4 Institute for Medical Microbiology, University of Basel, Basel, Switzerland; 5 Division of Infectious Diseases and Hospital Epidemiology, University Hospital Zürich, Zürich, Switzerland; 6 Division of Infectious Diseases and Hospital Epidemiology, University Hospital Basel, Basel, Switzerland; Drexel University College of Medicine, United States of America

## Abstract

**Background:**

In chronic HIV infection, antiretroviral therapy–induced normalization of CD4^+^ T cell counts (immune reconstitution [IR]) is associated with a decreased incidence of opportunistic diseases. However, some individuals remain at risk for opportunistic diseases despite prolonged normalization of CD4^+^ T cell counts. Deficient Epstein-Barr virus (EBV)-specific CD4^+^ T cell function may explain the occurrence of EBV-associated opportunistic malignancy—such as primary central nervous system (PCNS) lymphoma—despite recovery of absolute CD4^+^ T cell counts.

**Methods and Findings:**

Absolute CD4^+^ T cell counts and EBV-specific CD4^+^ T cell-dependent interferon-γ production were assessed in six HIV-positive individuals prior to development of PCNS lymphoma (“cases”), and these values were compared with those in 16 HIV-infected matched participants with no sign of EBV-associated pathology (“matched controls”) and 11 nonmatched HIV-negative blood donors. Half of the PCNS lymphoma patients fulfilled IR criteria (defined here as CD4^+^ T cell counts ≥500/μl blood). EBV-specific CD4^+^ T cells were assessed 0.5–4.7 y prior to diagnosis of lymphoma. In 0/6 cases versus 13/16 matched controls an EBV-specific CD4^+^ T cell response was detected (*p* = 0.007; confidence interval for odds ratio [0–0.40]). PCNS lymphoma patients also differed with regards to this response significantly from HIV-negative blood donors (*p* < 0.001, confidence interval for odds ratio [0–0.14]), but there was no evidence for a difference between HIV-negative participants and the HIV-positive matched controls (*p* = 0.47).

**Conclusions:**

Irrespective of absolute CD4^+^ T cell counts, HIV-positive patients who subsequently developed PCNS lymphoma lacked EBV-specific CD4^+^ T cell function. Larger, ideally prospective studies are needed to confirm these preliminary data, and clarify the impact of pathogen-specific versus surrogate marker-based assessment of IR on clinical outcome.

## Abstract 2

In a case-control study from the Swiss HIV cohort, Hess and colleagues report that T-helper responses against Epstein-Barr virus are specifically absent in patients developing CNS lymphoma.

## Editors' Summary

**Background.**

AIDS causes disease by inactivating the body's immune responses. Most severely affected are the white blood cells known as T lymphocytes, particularly the CD4^+^ T cells that recognize infection and enable other cells of the immune system to respond. Advanced HIV infection, marked by very low numbers of CD4^+^ cells, is associated with a variety of infections and tumors that are rarely seen in people with intact immune systems. People with advanced HIV who receive highly active antiretroviral treatment (HAART) tend to have increases in their CD4^+^ cell counts and lose their susceptibility to these so-called opportunistic infections and cancers. For several common opportunistic infections, it is considered safe to discontinue preventive antibiotics after a patient's total CD4^+^ cell count has returned to normal levels on HAART. Some treated individuals, however, will develop these conditions even after their CD4^+^ cell counts have returned to normal levels. The reason this happens is unclear.

**Why Was This Study Done?**

For several years, scientists have speculated that susceptibility to a given opportunistic infection might be due not simply to low total CD4^+^ cells, but to loss of the specific CD4^+^ cells that recognize the infection in question. If this theory is correct, then those individuals who develop an opportunistic condition after their total CD4^+^ cell counts return to normal might be missing the specific cells that respond to the microbe causing the condition. The researchers wanted to test this theory in HIV patients with a brain tumor called primary central nervous system lymphoma (PCNS lymphoma). The Epstein-Barr virus (EBV), which causes mononucleosis in the general population, has been shown to be a cause of PCNS lymphoma in people with AIDS.

**What Did the Researchers Do and Find?**

The researchers studied patients who developed PCNS lymphoma while enrolled in the Swiss HIV Cohort, an ongoing study that has enrolled more than 14,000 people. A large cohort was needed to address this question because PCNS lymphoma is uncommon, and indeed only six patients with a confirmed diagnosis were identified. Because they had been followed as part of the cohort study, these patients had given blood samples that could be tested in retrospect. Three of these patients had low CD4^+^ cell counts prior to lymphoma diagnosis and three had normal CD4^+^ cell counts, but CD4 responses specifically against EBV were absent or very low in all six patients before they were diagnosed with PCNS lymphoma. The researchers also studied a comparison group of cohort participants with comparable CD4^+^ cell counts but no PCNS lymphoma, and found that 13/16 of those participants did have CD4 responses to EBV.

**What Do These Findings Mean?**

These results support the idea that the action of EBV-specific CD4^+^ cells, rather than a given level of total CD4^+^ cells, is needed to prevent PCNS lymphoma. Because only a small number of cases were identified, this must be considered a preliminary result. Given the rarity of PCNS lymphoma, however, especially in people receiving HAART, it seems unlikely that a larger cohort will be available in the near future to provide a more definitive conclusion. Based on this result, it may be useful to perform similar studies of other opportunistic infections. If a “gap” in the CD4^+^ cell response can be shown to increase the risk of a specific condition, it may become appropriate to test specific CD4 responses before deciding to discontinue preventive treatment as CD4^+^ cell counts increase on HAART.

**Additional Information.**

Please access these Web sites via the online version of this summary at http://dx.doi.org/10.1371/journal.pmed.0040096.

- Read the accompanying Perspective by Mark Jacobson, MD
- The Swiss Cohort Study Web site contains information on related research projects
- The UCSF Center for HIV Information's HIV InSite includes resources on HIV immunology and opportunistic infections

## Introduction

The cardinal feature of HIV infection is depletion of CD4^+^ T cells from the circulation and from lymphoid tissue in most patients [1,2]. Immunodeficiency caused by CD4^+^ T cell depletion is associated with an increased risk for opportunistic diseases. The use of antiretroviral therapy (ART) has dramatically changed the course of HIV infection. ART-induced immune reconstitution (IR) is reflected by the fact that the incidence of opportunistic infections as well as several AIDS-defining malignancies decreases with treatment [3–7]. Specifically, the incidence of Epstein-Barr virus (EBV)-associated primary central nervous system (PCNS) lymphoma has dropped dramatically from 3–8 cases per 1,000 patient-years to about 1 case per 1,000 patient-years [8,9].

An increase of CD4^+^ T cell counts to levels ≥500/μl blood is used by some to define complete IR [10]. Among individuals with sustained IR, opportunistic diseases—including PCNS lymphoma—pose a particular challenge to both clinicians and immunologists. We lack controlled data on pathogen-specific CD4^+^ T cell immunity in patients developing PCNS lymphoma. Here, the existence of a large cohort of HIV-infected individuals allowed us to study, in a controlled setting, predisease virus-specific CD4^+^ T cell function in rare individuals who developed PCNS lymphoma.

## Methods

### Study Design

We performed a case-control study within the framework of the Swiss HIV Cohort Study, by searching the database for PCNS lymphoma occurring in patients later than 1997. Each HIV-positive patient who had developed PCNS lymphoma (“case”) was matched as available with two or three HIV-positive patients who had not developed PCNS lymphoma (“matched controls”) for: (i) age ± 5 y, (ii) sex, (iii) history of intravenous drug abuse, (iv) use of ART, (v) time of documented HIV positivity, (vi) duration of follow-up, and (vii) the closest available match of median and range of CD4^+^ T cell counts and HIV viral loads (Table 1). In both cases and matched controls, laboratory data were available every 3–6 mo. An additional control group consisted of 11 nonmatched HIV-negative individuals (“HIV-negative controls”). The study was IRB-approved and conducted according to the principles expressed in the Helsinki declaration, and written informed consent was obtained from all participants.

**Table 1:**
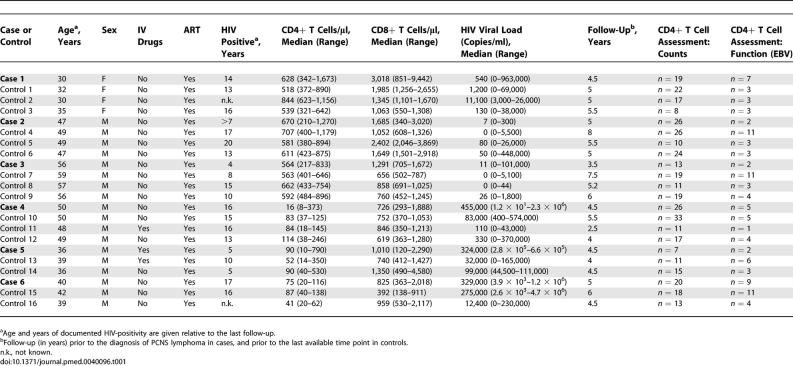
Patient Characteristics: Cases and Controls

### Isolation of Peripheral Blood Mononuclear Cells and Depletion of CD8^+^ T Cells

Peripheral blood mononuclear cells (PBMCs) were prepared by centrifugation through a density gradient on Histopaque-1077 (Sigma, http://www.sigmaaldrich.com). When using B cell lysate as the source of antigen, CD8^+^ T cells were depleted using anti-CD8 magnetic beads (Miltenyi Biotech, http://www.miltenyibiotec.com) following the manufacturer's protocol. Depletion was controlled for by FACScan-based enumeration, and was ≥98% complete (unpublished data).

### Assessment of EBV- and CMV-Specific CD4^+^ T Cell-Dependent Interferon-γ Production

Virus-specific interferon-γ (IFN-γ) production was determined by ELISPOT analyses as described [11,12]. Briefly, 96-well HTS ELISPOT plates from Millipore (http://www.millipore.com) were precoated overnight with 2 μg/ml of anti-IFN-γ mAb 1-D1K (Mabtech, http://www.mabtech.com), and washed six times with sterile PBS containing 1% FCS before use. After washing, 30 μl of R10 culture medium was added to each well to avoid drying of the membrane, and 100,000 viable cells per well added in 100 μl of R10. The EBV-specific CD4^+^ T cell response was assessed using as the source of antigen a set of peptides consisting of 33 HLA II-restricted optimal epitopes [11,12] and, in CD8^+^ T cell-depleted samples, EBV-infected B cell lysates (Virusys, http://www.virusys.com) [13]. For each participant at each time point the highest available SFC measurement (whether from peptide or B cell lysate assay) is reported. Cytomegalovirus (CMV)-specific IFN-γ production was assessed using a set of peptides consisting of 14 HLA II-restricted optimal epitopes (Table S1). Peptides (all synthesized at Massachusetts General Hospital, Boston, Massachusetts, United States) were added at a final concentration of 14 μg/ml, B cell lysates at a final concentration of 10 μg/ml. Peptides and B cell lysates were added in separate wells. The following controls were included in each experiment: Cells incubated with (i) medium alone, (ii) with a pool of 91 HLA I-restricted optimal EBV epitopes (as a functional control for the CD8^+^ T cell depletion) [11,12], and (iii) B cell lysate from EBV-naïve donors (Virusys). PHA was added at a concentration of 1.8 μg/ml to serve as positive control. As an additional control we tested an EBV-negative donor using peptides and (separately) EBV-infected B cell lysates. In this individual, both methods repeatedly yielded negative results (unpublished data).

Plates were incubated for 16 h at 37 °C with 5% CO_2_ before being developed. After washing with PBS, 100 μl of biotinylated anti-IFN-γ mAb 7-B6–1 (0.5 μg/ml, Mabtech) was added and plates incubated for 1 h at room temperature. Plates were washed again and incubated with a 1:2,000 dilution of streptavidin-coupled alkaline phosphatase (Streptavidin-ALP-PQ, Mabtech) for 1 h at room temperature in the dark. After further washing, IFN-γ production was detected as spots after 10–20 min incubation with nitroblue tetrazolium and 5-bromo-4-chloro-3-indolyl phosphate (BioRad, http://www.bio-rad.com). The color reaction was stopped by washing plates, and plates were air-dried before counting with an AID ELISPOT Reader Unit (Autoimmun Diagnostika, http://www.aid-diagnostika.com). Results are expressed as spot-forming cells (SFC) per million input cells. Thresholds for positive responses were determined as the mean plus three standard deviations of negative control wells.

### Determination of EBV-Specific Antibody Levels

EBV-VCA, and EBNA1-specific IgG antibody levels were determined using commercially available ELISA kits designed to detect IgG antibody bound to antigen-coated microtiter plates, following the manufacturer's protocol (ImmunoWell IgG test, GenGio, http://www.genbio.com).

### CMV Viral Load Determination

Viral DNA quantification was performed in serum or plasma samples using routine TaqMan real-time PCR technique (Applied Biosystems, http://www.appliedbiosystems.com).

### Statistical Analysis

If an individual patient's SFC measurements were 0 at more than 50% of available time points or, equivalently, if the patient's median over all available time points was 0, an EBV-specific CD4^+^ T cell response was defined as being absent. Association of “EBV-specific T cell response” with “outcome” (PCNS lymphoma or not) was examined with an exact Mantel-Haenszel test of conditional independence. As sensitivity analysis, the cut-off of 0 SFCs was replaced by 100. Also, the patient's outcome was modelled using exact conditional logistic regression with the logarithm of the patient's median (or mean) measurement of the EBV-specific CD4^+^ T cell response (plus 1 to deal with zero-measurements) as a covariate. Samples from HIV-negative volunteers were nonmatched, and comparisons were performed with Fisher's exact test. The Wilcoxon test was used to quantitatively compare median EBV-specific CD4^+^ T cell responses in HIV-negative controls with median CD4^+^ T cell responses in HIV-positive matched controls. All statistical tests were two-sided and performed at the 5% significance level. Statistical analyses were performed with SAS v9.1 software (SAS Institute, http://www.sas.com).

## Results

In a search of the Swiss HIV Cohort Study database, six HIV-positive individuals who developed PCNS lymphoma were identified (cases 1–6). Diagnosis was established via biopsy in cases 1, 2, 3, and 5, and via imaging, detection of EBV in cerebrospinal fluid, and exclusion of alternate diagnoses in cases 4 and 6. EBV-specific antibodies were detected in all PCNS lymphoma patients more than 2.5 years prior to diagnosis of lymphoma, and in all matched controls. The search criteria applied for selection of control patients are described in the Methods, and patient characteristics are summarized in Table 1.

**Figure 1:**
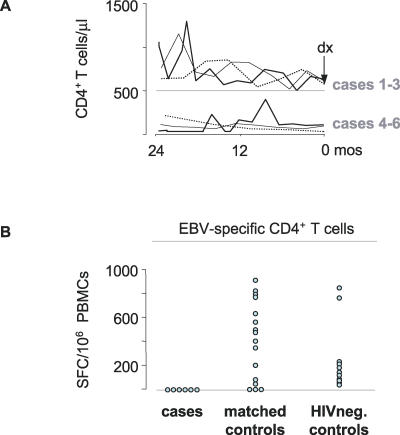
Absolute CD4^+^ T Cell Counts versus EBV-Specific CD4^+^ T Cell Function (A) CD4^+^ T cell counts from cases 1–6 throughout two years prior to diagnosis of PCNS lymphoma are shown. Note that in cases 1–3 CD4^+^ T cell counts remained continuously ≥500/μl. (B) Ex vivo assessment of EBV-specific CD4^+^ T cell-dependent IFN-γ production in HIV-positive patients (cases 1–6) prior to diagnosing PCNS lymphoma (left), matched control participants (middle), and HIV-negative healthy controls (right). Median values of the maximum SFC measurement at each available time point are shown for cases and matched controls, single SFC values for the HIV-negative nonmatched study population. Note the lack of EBV-specific CD4^+^ T cell activity in progressors to PCNS lymphoma. In contrast, HIV-infected patients with no sign of EBV-associated pathology (but a wide range of absolute CD4^+^ T cell counts) did not significantly differ from HIV-negative healthy control participants.

Three of the six PCNS lymphoma patients fulfilled IR criteria (cases 1–3) (Figure 1A and Table 1). EBV-specific CD4^+^ T cell-mediated IFN-γ production was assessed covering a range of 0.5–4.7 y prior to diagnosis of lymphoma. The n-number of time points at which functional assays were performed in each patient is listed in Table 1. In 0/6 cases versus 13/16 matched controls an EBV-specific CD4^+^ T cell-response was detected (*p* = 0.007; odds ratio 0, confidence interval [0–0.40]) (Figure 1B, left and middle). Sensitivity analyses and exact conditional regressions were also significant (*p* < 0.05 for all analyses). Absolute CD4^+^ T cell counts in samples tested from each of the controls were ≤ absolute CD4^+^ T cell counts of samples tested from cases, thus excluding the possibility that the observed disparities were driven by differences in CD4^+^ T cell counts rather than by pathogen-specific CD4^+^ T cell function. The maximum SFC measurement at each available time point for each case and matched control patient is listed in Table S2, and graphically presented in Figure S1.

We also assessed the EBV-specific CD4^+^ T cell-mediated IFN-γ production in 11 randomly selected HIV-negative blood donors (Figure 1B, right). Not surprisingly, PCNS lymphoma patients also differed in this measurement from these HIV-negative controls (*p* < 0.001, odds ratio 0, confidence interval [0–0.14]). The median EBV-specific CD4^+^ T cell-response in HIV-positive and HIV-negative controls was 403 (range 0–905) and 130 (range 40–840), respectively, and there was no evidence of a difference in these responses (*p* = 0.47).

Aiming at distinguishing generally depressed CD4^+^ T cell activity from specifically decreased anti-EBV responses in the participants who developed PCNS lymphoma, we tested the CMV-specific CD4^+^ T cell-response in CMV-positive cases (*n* = 4), and compared it to CMV-positive controls (*n* = 4). Responses were similar, with medians of 540 SFC/10^6^ PBMCs (range 30–1,000 SFC/10^6^ PBMCs) and 280 SFC/10^6^ PBMCs (range 55–1,000 SFC/10^6^ PBMCs), respectively. In CMV-negative cases and controls no CMV-specific CD4^+^ T cell response was detected.

To further test for the specificity of a possible immune deficit, CMV viral loads were assessed longitudinally in these same CMV-positive cases and controls. In each patient four to 11 samples were tested (58 PCR assays in total). CMV DNA was detected in only two of 29 versus one of 29 samples in CMV-positive patients versus control participants, respectively. Thus, based on circulating DNA-levels, CMV was well controlled in both cases and controls.

## Discussion

Although PCNS lymphoma can develop in HIV-negative individuals, it much more often occurs in severely immunosuppressed HIV-infected persons (CD4^+^ T cell counts <50/μl). Intriguingly, since the introduction of ART, PCNS lymphoma has also been reported in individuals with CD4^+^ T cell counts over 200/μl or even over 500/μl [14,15].

Here we observed—irrespectively of absolute CD4^+^ T cell counts—an almost complete lack of EBV-specific CD4^+^ T cells in HIV-positive patients who progressed to PCNS lymphoma. Inversely, even with low CD4^+^ T cell counts, EBV-specific CD4^+^ T cells were detectable in individuals with no sign of EBV-associated pathology. Similar CMV-specific CD4^+^ T cell responses and (largely) undetectable CMV replication suggest that CMV-specific immunity was adequate in both cases and controls.

The lack of EBV-specific CD4^+^ T cell function irrespective of absolute CD4^+^ T cell counts likely represents a more general phenomenon. Case reports and a small case-control study have reported clinically significant CMV and Pneumocystis jiroveci infection in individuals with elevated absolute CD4^+^ T cell counts, and pathogen-specific CD4^+^ T cell deficiency has been suggested to persist in these patients [16–20]. The fact that seemingly successful ART does not necessarily allow for the reappearance of pathogen-specific CD4^+^ T cells is consistent with a recent report showing that during acute simian immunodeficiency virus infection, up to 60% of all CD4^+^ T memory cells throughout the body are irreversibly lost [2]. It thus seems possible that, depending on the pre-depletion breadth and magnitude of a given pathogen-specific immune response, untreated acute HIV infection has the capacity to remove most (if not all) CD4^+^ T cells with specificity for a particular pathogen. Such thorough depletion may then be largely unresponsive to long-term control of HIV replication by ART and subsequent replenishment of absolute CD4^+^ T cell numbers.

Absence of EBV-specific CD4^+^ T cell effector function and/or help for cytotoxic CD8^+^ T cells may provide an immunological basis for development of PCNS lymphoma in HIV-infected individuals [21–23]. Larger, ideally prospective studies are needed to confirm these preliminary data, and to clarify the impact of pathogen-specific versus surrogate marker-based assessment of IR on clinical outcome.

## Supporting Information

Figure S1 Ex Vivo Assessment of EBV-Specific CD4^+^ T Cell-Dependent IFN-γ Production in Positive Patients Prior to Diagnosing PCNS Lymphoma and in Matched-Control Participants Shown are progressors (cases) before diagnosis (left) and matched control participants (right). Each dot represents the maximum SFC result for a given participant at a given time point. Overlapping dots are distributed on the x-axis to remain separated.
(32 KB PDF)

Table S1 HLA II-Restricted Epitopes Used to Detect CMV-Specific CD4^+^ T Cells (33 KB DOC)

Table S2 EBV-Specific CD4^+^ T Cell-Mediated IFN-γ Production: Maximum SFC Values at Each Time Point Tested for Cases and Matched Controls (64 KB DOC)

## Abbreviations

ART: antiretroviral therapy
CMV: cytomegalovirus
EBV: Epstein-Barr virus
IFN: interferon
IR: immune reconstitution
PBMC: peripheral blood mononuclear cell
PCNS-lymphoma: primary central nervous system lymphoma
SFC: spot-forming cells
