# Supplementary figures and images for: HIV Patients Developing Primary CNS Lymphoma Lack EBV-Specific CD4+ T Cell Function Irrespective of Absolute CD4+ T Cell Counts
Source: PLoS Med. 2007 Mar 27;4(3):e96. doi: 10.1371/journal.pmed.0040096 (PMC1831733; doi:10.1371/journal.pmed.0040096)

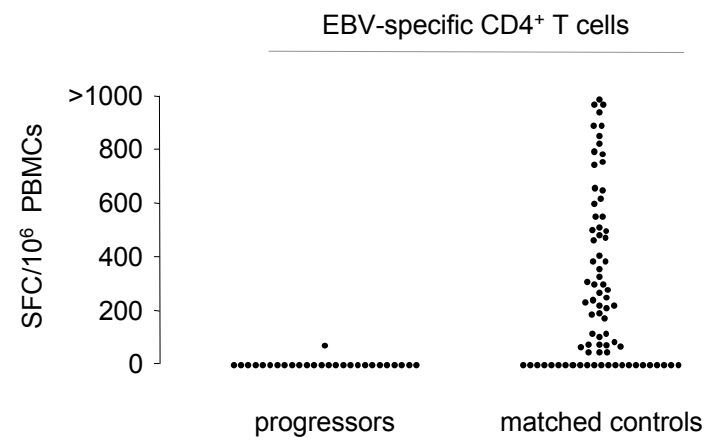

**Supplementary Figure 1**

Supplement: Figure S1 — Shown are progressors (cases) before diagnosis (left) and matched control participants (right). Each dot represents the maximum SFC result for a given participant at a given time point. Overlapping dots are distributed on the x-axis to remain separated. (32 KB PDF) [file pmed.0040096.sg001.pdf]
