# Supplementary material for: HIV Patients Developing Primary CNS Lymphoma Lack EBV-Specific CD4+ T Cell Function Irrespective of Absolute CD4+ T Cell Counts
Source: PLoS Med. 2007 Mar 27;4(3):e96. doi: 10.1371/journal.pmed.0040096 (PMC1831733; doi:10.1371/journal.pmed.0040096)
Supplement: Table S1 — (33 KB DOC) [file pmed.0040096.st001.doc]

| CMV protein | aminoacids | position |
| --- | --- | --- |
| CMVpp65-N1 | PSLILVSQYTPDSTP | aa 53-67 |
| CMVpp65-N2 | LVSQYTPDSTPPCHRG | aa57-71 |
| CMVpp65-N3 | MSIYVYALPLKMLNI | aa109-123 |
| CMVpp65-N4 | EPDVYYTSAFVPTK | aa177-191 |
| CMVpp65-N5 | AHELVCSMENTRATK | aa201-215 |
| CMVpp65-N6 | SGKLFMHVTLGSDVE | aa237-251 |
| CMVpp65-N7 | DLTMTRNPQPFMRPH | aa253-267 |
| CMVpp65-N8 | GKISHIMLDVAFTS | aa285-299 |
| CMVpp65-N9 | SIPGLSISGNLLMNG | aa309-323 |
| CMVpp65-N10 | GVMTRGRLKAESTVA | aa449-463 |
| CMVpp65-N11 | RGRLKAESTVAPEED | aa453-467 |
| CMVpp65-N12 | AGILARNLVPMVATV | aa489-503 |
| CMVpp65-N13 | ARNLVPMVATVQGQN | aa493-507 |
| CMVpp65-N14 | KYQEFFWDANDIYRI | aa509-523 |

# Supplementary Table 1

HLA II-restricted epitopes used to detect CMV-specific CD4^+^ T cells
