# Supplementary material for: HIV Patients Developing Primary CNS Lymphoma Lack EBV-Specific CD4+ T Cell Function Irrespective of Absolute CD4+ T Cell Counts
Source: PLoS Med. 2007 Mar 27;4(3):e96. doi: 10.1371/journal.pmed.0040096 (PMC1831733; doi:10.1371/journal.pmed.0040096)
Supplement: Table S2 — (64 KB DOC) [file pmed.0040096.st002.doc]

|  |  |  |  |  |  |
| --- | --- | --- | --- | --- | --- |
| case 1 | case 2 | case 3 | case 4 | case 5 | case 6 |
| 0 | 0 | 0 | 0 | 0 | 0 |
| 0 | 0 | 0 | 0 | 0 | 0 |
| 0 |  |  | 0 |  | 0 |
| 0 |  |  | 0 |  | 0 |
| 0 |  |  | 0 |  | 0 |
| 0 |  |  |  |  | 0 |
| 0 |  |  |  |  | 0 |
|  |  |  |  |  | 0 |
|  |  |  |  |  | 70 |
|  |  |  |  |  |  |
| ctrls for c1 | ctrls for c2 | ctrls for c3 | ctrls for c4 | ctrls for c5 | ctrls for c6 |
| 0 | 0 | 53 | 0 | 0 | 30 |
| 0 | 0 | 30 | 0 | 0 | 0 |
| 180 | 0 | 0 | 90 | 175 | 290 |
|  | 0 | 0 | 0 | 210 | 30 |
|  | 0 | 0 | 0 | 300 | 60 |
|  | 0 | 0 |  | 210 | 60 |
|  | 50 | 900 |  |  | 0 |
|  | 0 | 380 |  |  | 100 |
|  | 50 | 220 |  |  | 0 |
|  | 160 | 270 |  |  | 260 |
|  | 240 | 230 |  |  | 0 |
| 480 | 500 | 400 | 480 | 459 | 350 |
| 900 | 790 | 550 |  | 950 | 510 |
| 800 | 860 | 660 |  | 620 | 70 |
|  |  |  |  |  | 320 |
| 470 | 750 | 980 | 290 |  |  |
| 100 | 760 | 980 | 380 |  |  |
| 550 | 1000 | 650 | 200 |  |  |
|  |  | 830 | 600 |  |  |

# Table S2

The highest available, background-subtracted SFC-values/10^6 PBMCs of all cases (c1-6) and their controls (ctrls) are shown, with each SFC-value representing a different time point. Measurements obtained in a given individual are shown within an individual cell.
